# Data-driven inference of network connectivity for modeling the dynamics of neural codes in the insect antennal lobe

**DOI:** 10.3389/fncom.2014.00070

**Published:** 2014-08-13

**Authors:** Eli Shlizerman, Jeffrey A. Riffell, J. Nathan Kutz

**Affiliations:** ^1^Department of Applied Mathematics, University of WashingtonSeattle, WA, USA; ^2^Department of Biology, University of WashingtonSeattle, WA, USA

**Keywords:** data-driven modeling, reduced dynamics, olfactory neural coding, neuronal networks, odor discrimination, contrast enhancement, insect olfaction

## Abstract

The antennal lobe (AL), olfactory processing center in insects, is able to process stimuli into distinct neural activity patterns, called olfactory neural codes. To model their dynamics we perform multichannel recordings from the projection neurons in the AL driven by different odorants. We then derive a dynamic neuronal network from the electrophysiological data. The network consists of lateral-inhibitory neurons and excitatory neurons (modeled as firing-rate units), and is capable of producing unique olfactory neural codes for the tested odorants. To construct the network, we (1) design a projection, an odor space, for the neural recording from the AL, which discriminates between distinct odorants trajectories (2) characterize scent recognition, i.e., decision-making based on olfactory signals and (3) infer the wiring of the neural circuit, the connectome of the AL. We show that the constructed model is consistent with biological observations, such as contrast enhancement and robustness to noise. The study suggests a data-driven approach to answer a key biological question in identifying how lateral inhibitory neurons can be wired to excitatory neurons to permit robust activity patterns.

## 1. Introduction

In the olfactory system, neural codes take the form of spatial firing-rate (FR) patterns exhibited by the output neurons of the neural processing unit, the *antennal lobe* (AL) in insects and *olfactory bulb* (OB) in mammals (Laurent, 1999, 2002; Stopfer and Laurent, 1999; Galizia and Menzel, 2000; Riffell et al., 2009a). They were established by the application of standard data analysis techniques, e.g., Principal Components Analysis (PCA), to the time series of FR responses recorded from the output neurons (Broome et al., 2006; Harris et al., 2011). The success of these methods indicates that the response of cell assemblies is indeed *low-dimensional* so that for each individual stimulus a unique trajectory in a low-dimensional subspace is identified.

With these discoveries, it is intriguing to understand how sensory neural networks are designed to produce such behavior. Specifically, why do the encoding dynamics appear to be **robust** even for noisy stimuli? and what is the network **architecture** capable to produce these patterns (Wilson, 2008; Nagel and Wilson, 2011)? For the first question, investigations suggest that cell assemblies maintain several mechanisms for shaping the correct output response. One such mechanism is known to be *lateral inhibition* (Laurent, 1999; Egger et al., 2003), where both inhibitory and excitatory neurons receive common input and interact to mediate the response of excitatory neurons. A hallmark of lateral inhibition is *contrast enhancement*, which signature is an increase in signal to noise ratio, such that the amplitude or frequency of the response is easily distinguished from the response to random stimuli (Laughlin and Osorio, 1989; Yokoi et al., 1995; Cook and McReynolds, 1998; Olsen and Wilson, 2008; Wilson, 2008).

For the question of determining the network architecture that produces the neural codes it is required to model the actual network of neurons responsible for the encoding. The modeling procedure involves reconstruction of the *network wiring*, i.e., modeling individual neuron dynamics and their network interactions (*connectome*) (Seung, 2011; Jbabdi and Behrens, 2012). However, the connectome of different sensory neuronal networks may vary. For example, in vision, the retinal ganglion cells are ordered such that locally neighboring cells are responsive to neighboring parts of the visual stimulus, termed a *retinotopic map* (Bock et al., 2011). In olfaction, output neurons are also selective for certain odorant stimuli, providing a *chemotopic map*. However, the neighboring output neurons are not necessarily similar in their tuning to specific chemicals. Instead lateral inhibition mediates and shapes the responses of the output neurons, resulting in an *effective chemotopic map* between the input and the output cells (Cleland and Linster, 2005; Linster et al., 2005; Silbering and Galizia, 2007; Reisenman et al., 2008). Resolving this mapping is critical for determining how neurons process chemical information.

Inspired by the dimension reduction results and in order to find the mechanism responsible for the low dimensional dynamics several approaches were proposed to model the underlying neuronal network (reviewed in detail in Buckley and Nowotny, 2012). Afraimovich et al. (2004) used the top–down approach to construct the connectivity by restricting the network to have particular dynamics governed by low-dimensional homoclinic orbits deriving a ‘winnerless’ network able to produce robust transient dynamics in low-dimensional space (see also Rabinovich et al., 2001, 2006). Alternatively, Linster et al. (2005) used a bottom–up approach to derive a qualitative model for the honeybees AL where each neuron was modeled as a FR unit and the connectivity chosen to be inhibitory and drawn from a distribution that matches experimental knowledge (e.g., connecting pairs proportional to the similarity of their odor-response profiles). The model showed correspondence with experimental data when compared with calcium imaging dynamics suggesting that functionally organized inhibitory network, as opposed to anatomically structured network (local), all-to-all or random inhibitory network, best reproduces the input-output function of the AL. The use of firing-rate models was later justified by Buckley and Nowotny (2011) where it was shown that a network of Hodgkin–Huxley neurons can be reduced to firing-rate models and that stable fixed point dynamics are the most consistent with the FR time series data. While these works show that the AL is capable of extracting low dimensional features and can be modeled using firing rate units, the wiring of lateral inhibition within the models is set randomly. Therefore, the structural properties of the network that permit neural codes still remain unresolved (Rabinovich et al., 2008). Without resolving the network connectivity one cannot understand the observed features of odor processing such as contrast enhancement.

Electrophysiological recordings that sample the output from the AL could be potentially useful to infer candidates for such wirings. The most suitable approach for solving this ‘inverse problem’ would be the top–down approach, since it attempts to construct a low-dimensional model and establish the underlying mechanisms that determine network units and connectivity. However, it is currently unknown how to calibrate the low dimensional model using multi-neuron recordings (Mazor and Laurent, 2005; Rabinovich et al., 2008; Buckley and Nowotny, 2012). To overcome this difficulty, we propose the dynamical dimension reduction method that takes the *top–down approach in conjunction with multineuron recordings*. With this methodology we model the AL in the *Manduca sexta* moth a well-characterized physiological and behavioral experimental neural system in olfaction (Reisenman et al., 2008; Riffell et al., 2009a). The outcome of the approach is a high-dimensional system that exhibits low-dimensional dynamics. The method is fundamentally different than standard top-down approaches, as it does not determine parameters by simulation and fitting, which are biased by the simulations performed (e.g., choice of initial conditions), fitting of time-dependant signals and comparison metric. Instead, it projects a high-dimensional dynamical system onto orthogonal modes to be derived from data. This step is implicit and achieves a projected low dimensional system that is generic. As such it could be applicable for modeling the AL across different species and other neuronal networks. In the following step the projected dynamical system is matched with the conjectured low-dimensional dynamical system. We show that in the case of fixed points the matching can be formulated as a convex optimization problem. The system becomes explicit when the neural codes obtained from recordings are plugged-in and the optimization problem is solved to infer the wiring. For the AL, the matching is based on the characteristic that the projected dynamics onto the neural codes exhibit a trajectory toward a well separated fixed point for each stimulus, a consistent feature in experimental observations and used for modeling in Buckley and Nowotny (2012). Inputting the experimental neural codes as orthogonal modes and solving a minimization problem infers a suggested network wiring of the AL capable to encode given stimuli. The model is then compared with the experimental dynamics for consistency. Our results show that the wirings obtained using such an approach produce neural codes that are unsusceptible to noise and thus suggest that the introduced methodology can assist in resolving the architecture of the AL and circuit-level properties.

## 2. Materials and methods

### 2.1. Data driven top–down modeling approach

The neural cell assemblies participating in the processing of olfactory information in the AL are the receptor cells (RNs) that carry the input from the environment, the projection (output) neurons (PNs), and local interneurons (LNs) (reviewed by Hildebrand and Shepherd, 1997; Hansson and Anton, 2000; Martin et al., 2011). We model the network by three vectors $\vec{x},\vec{y},$ and $\vec{z}$, where each element in the vector represents a neuron and modeled by a firing-rate unit (Linster et al., 2005; Capurro et al., 2012; Chong et al., 2012). The three vectors correspond to the three anatomical groups RNs, PNs, and LNs, respectively:

(1) $$\dot{\vec{x}}=-\vec{x}+\vec{J},$$

(2) $$\dot{\vec{y}}=-\beta \vec{y}+{\left[A\vec{x}-B\vec{z}\right]}^{+},$$

(3) $$\dot{\vec{z}}=-\gamma \vec{z}+{\left[C\vec{x}-E\vec{z}\right]}^{+}.$$

The input into the PNs and LNs is modulated by a standard linear threshold function denoted by [.]^+^, as in Linster et al. (2005) and Buckley and Nowotny (2012). **Figure 2A** illustrates the threshold function used here. The vector $\vec{J}$ is the external input into the RNs which is driven by the chemosensory processes in the antenna.

In the deterministic version of this model, where the input is either constant or time dependant, the dynamics can be intuitively described. Specifically, when there is significant input into the population of receptor neurons ($\vec{x}$), these neurons lock onto the driving input $\vec{J}$ (Buckley and Nowotny, 2011, 2012). In the case of constant input, the receptor population will converge to a fixed point ${\vec{x}}_{0}=\vec{J}$. This in turn excites both the projection neurons ($\vec{y}$) and the interneuron populations ($\vec{z}$). A *meaningful* input should excite a spatial *stable* pattern ${\vec{y}}^{P}$ in the projection neurons. The stable spatial patterns ${\vec{y}}^{P}$ are thought of as library elements which encode various recognized odorants. Note that the pattern is not necessarily equal to the input, i.e., ${\vec{y}}^{P}\neq \vec{J}$.

Our goal is to understand how the network in Equations (1–3) can be made capable to produce stable patterns and discriminate between them. Particularly, we would like to find a network connectome, consisting of the connectivity matrices *A, B, C*, and *E*, that enhances the components in the input that correspond to recognized patterns (${\vec{y}}^{P}$) and inhibits other remaining components. In practice, the structure of the connectivity matrices *A* and *C* is local and can be obtained from anatomical experimental knowledge, while the structure of the matrices *B* and *E* is mostly unknown.

The method *dynamical dimension reduction* that we introduce in this work provides a procedure to construct the unknown matrices *B* and *E*. The first step in the method is to obtain population encoding vectors (orthogonal patterns ${\vec{y}}^{P}$) from the electrophysiological recordings of PN neurons. We then show that a projection of the PN dynamical equations, Equation (2), onto the population encoding vectors provides a division of these equations into two models: a reduced model for the dynamics of population vectors and a model for the dynamics of remaining patterns. Separating the system into two models allows us to impose constraints on the dynamics of each model. Particularly, we require stable patterns in the reduced model and rapid decay of the remainder. We show that these requirements form a convex minimization problem which solution is the unknown connectome.

The projection is done as follows. If the system does not saturate, then the excitable regime can be modeled by a linear version of Equations (2–3) in which the brackets from the saturation terms are removed. Additionally, if the $\vec{x}$ dynamics are fast in comparison to those of $\vec{y}$ and $\vec{z}$ (RNs drive the response in LNs and PNs) (Geiger et al., 1997; Meyer et al., 2013), then $\vec{x}$ can be replaced by the input $\vec{J}$, i.e., its fixed point, and we derive the following system

(4) $$\frac{d\vec{y}}{dt}=-\beta \vec{y}+A\vec{J}-B\vec{z},$$

(5) $$\frac{d\vec{z}}{dt}=-\gamma \vec{z}+C\vec{J}-E\vec{z}.$$

In this system, the vector $\vec{y}(t)$ describes the dynamics of the coefficients of a standard basis [*y_i_*(*t*) is the dynamics of *i*-th PN neuron]. However, we are interested in determining the dynamics of the observed patterns. From this representation, it is not immediately clear how to conclude which coding patterns in $\vec{y}$ appear while others do not, and what kind of connectivity matrices support such formations. Thus, the next step in our analysis is to decompose the system into encoding patterns and the remainder. For such a decomposition, we assume that there is a library matrix *L* of observed patterns $L=\{{\vec{y}}_{1}^{P},\ldots ,{\vec{y}}_{l}^{P}\}$. We take into account that the library is a semi-positive matrix and we normalize each column vector (pattern) in the matrix. We then transform the matrix to an *orthonormal matrix O^P^*. In this matrix, each column vector is called a *population encoding vector* and represents neurons and their expected firing-rates evoked by a particular input-key. The transformation to the orthonormal matrix is achieved by applying a threshold and a maximum rule on each element *l_ij_* of the matrix *L*. Thereby each element *o^P^_ij_* in the matrix *O^P^* is defined as follows

$$o_{ij}^{P}=U_{1}(l_{ij})=\{\begin{array}{l} \begin{array}{l} l_{ij}\text{ if }l_{ij}=\max({\vec{l}}_{i})\geq \tau .\\ 0\text{ otherwise} \end{array} \end{array}$$

where τ is the threshold value (chosen as τ = 0.07 in **Figure 5**). This construction results in a matrix with a single positive element in each row vector or a zero row vector, such that the system is effectively made orthogonal. The zero row vectors indicate PN neurons that do not substantially contribute to any of the patterns and thus these neurons will be considered to belong to the *remainder* vector. To construct the remainder vector, ${\vec{o}}^{R}$, we define the transformation *U_2_*

$${\vec{o}}^{R}=U_{2}(U_{1}(l_{ij}))=\{\begin{array}{l} \begin{array}{l} 1\text{ if }\max({\vec{l}}_{i})=0\\ 0\text{ otherwise}. \end{array} \end{array}$$

that assigns the value of unity if the corresponding row in *O^P^* that is a zero vector. As a final step we normalize ${\vec{o}}^{R}$ and augment the matrix *O^P^* with the vector ${\vec{o}}^{R}$ to create the matrix *O*:

$$L={\left[\begin{array}{ccc} \vdots & & \vdots \\ {\vec{y}}_{1}^{P} & \cdots & {\vec{y}}_{l}^{P}\\ \vdots & & \vdots \end{array}\right]}_{N\times l}\to^{U}O={\left[\begin{array}{ccc} \begin{array}{c} \vdots \end{array} & \vdots & \vdots \\ {\vec{o}}_{1}^{P} & \cdots {\vec{o}}_{l}^{P} & {\vec{o}}^{R}\\ \vdots & \vdots & \vdots \end{array}\right]}_{N\times l+1}.$$

This allows us to describe the dynamics of PNs with the following low rank decomposition

(6) $$\begin{array}{l} \vec{y}(t)=p_{1}(t){\vec{o}}_{1}^{P}+\ldots +p_{l}(t){\vec{o}}_{l}^{P}+r(t){\vec{o}}^{R}\\ \;=O\left(\begin{array}{c} \begin{array}{l} p_{1}(t)\\ \;\vdots \\ p_{l}(t)\\ \;r(t) \end{array} \end{array}\right)=O\vec{p} \end{array}$$

Here we multiply each of the population vectors (stationary) by a dynamical coefficient *p_j_*(*t*) and the remainder population vector by *r(t)*. To derive the equations for the dynamics of the coefficients $\vec{p}(t)$, we substitute the decomposition of Equation (6) into Equation (4) and multiply the equations for $\vec{y}$ by the transpose matrix *O^T^* and use the fact that for semi-orthogonal matrices *O^T^O* = *I*. Thus,

(7) $$\begin{array}{l} \frac{d\vec{p}}{dt}=-\beta \vec{p}+O^{T}(A\vec{J}-B\vec{z}),\\ \frac{d\vec{z}}{dt}=-\gamma \vec{z}+C\vec{J}-E\vec{z}. \end{array}$$

This projection technique is based on the Proper Orthogonal Decomposition method introduced in Sirovich (1987, 1996) and applied to reduction of neuronal networks in Shlizerman et al. (2012).

In this section we consider the case where the input is time-independent and in the Results section explore the system dynamics with time dependent and noisy inputs. Since the dynamics in $\vec{z}$ are independent of the dynamics in $\vec{p}$, we can solve the second equation in Equation (7) for a fixed point $d\vec{z}/dt=0$

$${\vec{z}}_{0}={\tilde{E}}^{-1}C\vec{J},\;\;\;\;\;\;\tilde{E}=E+\gamma I.$$

Then plugging-in into the first equation the expression of the fixed point we receive

(8) $$\begin{array}{l} \frac{d\vec{p}}{dt}=\tilde{M}\vec{p}+{\vec{J}}^{eff}\\ \tilde{M}=-\beta I,\;\;{\vec{J}}^{eff}=O^{T}(A-B{\tilde{E}}^{-1}C)\vec{J}. \end{array}$$

The resulting reduced system is a linear inhomogeneous system of ODEs. For constant inputs, LNs will eventually equilibrate to the fixed point ${\vec{z}}_{0}$, determined by the values of γ and *E*. For pulsed stimuli, which are often considered in experiments (e.g., the duration of stimulation used here is 200 ms), LNs typically respond to the onset of stimulus with bursting and release fast GABA-A transmitters (within 1–2 ms) (Christensen et al., 1998) and later with slower GABA-B transmitters (100 ms) and are represented by the matrix *E*. Since there is a separation of time-scales between these types of transmitters, as a first order approximation the matrix *E* represents fast connections, although both timescales can be incorporated.

The system in Equation (8) has terms that include the parameter $\vec{p}$ (multiplied by $\tilde{M}$) and non-homogeneous terms that are the effective input. Note that since there is no input from $\vec{y}$ into $\vec{z}$ (PN to LN) in Equations (4–5), the homogeneous term is multiplied by a diagonal matrix $\tilde{M}$. The matrix $\tilde{M}$ has only negative eigenvalues (λ_*i*_ = −β) and thus by Lyapunov's stability theorem the model in Equation (8) is globally asymptotically stable, i.e, the system will always converge to a stable equilibrium ${\vec{p}}_{0}=(1/\beta ){\vec{J}}^{eff}$ (Gajic and Lelic, 1996), see **Figure 2C**. In systems which have additional input from the $\vec{y}$ population into the $\vec{z}$ population, the matrix $\tilde{M}$ will involve non-diagonal terms that express interactions of the patterns. For such wirings it should be verified that the system is stable, i.e., the dynamics are as in **Figure 2C**, by solving the Lyapunov equation that will involve the connectivity matrices (Gajic and Lelic, 1996). The solution of the equation, if exists, will impose constraints on the configuration of the connectivity matrices such that the fixed point is stable. These constraints will be added to the optimization problem (11).

While the stability theorem assures that the dynamics of the patterns converge to an equilibrium, it does not guarantee separation of equilibria, which is required for a robust encoding-decoding system. Moreover, the *matrices B and Ẽ are unknown*, both in theory and in practice. For that purpose we need to calibrate the effective input into the population encoding vectors. Following the same procedure as for the output patterns we construct an *orthogonal library matrix, J*_0_, for the input keys. Then the calibration is reduced to solving the following system of underdetermined equations

(9) $$O^{T}(A-B{\tilde{E}}^{-1}C)J_{0}=W.$$

with the prescribed matrix *W* of dimensions (*l* + 1) × (*l* + 1) representing the calibration, and *B* and Ẽ are the unknown matrices. Essentially, this is a linear system of equations with a specified right hand side matrix *W* where the matrix elements of *W* determine physiologically relevant characterization of the importance of various odors. This is a highly undetermined set of equations that allows for an infinite number of solutions, i.e., there are an infinite number of ways to specify *B* and Ẽ. Imposing further biophysical constraints could allow to obtain a unique biophysical solution.

Each row in *W* encodes the effect of the different input keys, including the remainder, on a particular population encoding vector. For example, the element on the *i*-th row and *k*-th column, *w_i,k_*, defines how ${\vec{J}}_{k}$ excites or inhibits *p_i_*(*t*). The elements of *W* are set as follows

(10) $$W=\left[\begin{array}{c} \begin{array}{l} \underline{\;\ddots \;}\\ \underline{\;w_{i,i}\;w_{i,k}\;w_{i,l+1}\;} \end{array}\\ \underline{\;\ddots \;}\\ \begin{array}{ll} w_{l+1,1}\; & w_{l+1,k}\;w_{l+1,l+1} \end{array} \end{array}\right].$$

The diagonal element on the *i*-th row, *w_i,i_*, defines how ${\vec{J}}_{i}$ affects *p_i_*(*t*), its corresponding population encoding vector, and has to be set as positive (excitatory). The input from the other keys, ${\vec{J}}_{k}$, *k* ≠ *i*, is encoded by *w_i,k_* and can be set 0 or negative. The input from the last key is the input from the remainder and is encoded by *w_i,l_*_+ 1_. The value of this element should be strictly set to 0, such that the remainder does not have excitatory or inhibitory effect on the population encoding vector. The last row in *W* denotes the input into the remainder and thereby the elements, except the diagonal element on that row should be always negative. See the caption of **Figure 2** for a possible configuration of the matrix *W*.

When *A* and *C* are known matrices, then the calibration is accomplished by solving an inverse problem to find the matrices Ẽ and *B* that satisfy these equations. Notice that the equations are underdetermined, i.e., the dimensions of *W* are much lower than of *B*Ẽ^−1^, indicating that the matrices *B* and Ẽ that satisfy Equation (9) are non-unique. To find the appropriate candidates for the matrices, we reformulate the inverse problem as a minimization problem

(11) $$\begin{array}{l} \text{minimize}\;\;||O^{T}(A-B{\tilde{E}}^{-1}C)J_{0}-W|{|}_{Fr}\\ \text{ subject}\;\text{to}\;\;\;\;\text{B},\text{E}\geq \text{ 0},\;\;\;\;\;\;\;\; \end{array}$$

where ‖·‖ is the Frobenius matrix norm. When the lateral connections between PNs and LNs are exclusively inhibitory the matrices *B*, Ẽ are non-negative. When one of the matrices is set to particular wiring (e.g., Ẽ is random) we need to determine only one matrix and the minimization problem is a semi-definite convex minimization. When there are excitatory lateral connections or the zero minimum cannot be attained, the semi-definite constraint is relaxed. Another possibility for negative terms in *B* and Ẽ is when the input keys and the output codes differ from each other in dimensions. Indeed, the matrices *B* and Ẽ permute the lateral effect of the interneurons to support such a coding scheme. Due to many degrees of freedom in the problem, additional constraints can be added. For example we can restrict the magnitudes of the elements in *B* and Ẽ not to exceed a particular value. Moreover, the calibration is particular to the choice of the matrices *A* and *C* (see an example in **Figure 2B**). To solve the minimization problem (11) or its variants, we employ the disciplined convex optimization package CVX implemented in MATLAB Grant and Boyd (2011).

For input keys being identical to the output population vectors, i.e., *J*_0_ ≡ *O*, the calibration creates a system that for a significant magnitude of one of the input keys, noise, and other population encoding vectors will be suppressed to allow for a decoding of the input-key (see **Figure 4**). Effectively this is a mechanism that produces contrast enhancement as we discuss in the Results section.

### 2.2. Electrophysiological preparation and stimulation

*Manduca sexta* L. (Lepidoptera: Sphingidae) male larvae were obtained from the *Manduca*-rearing facility of the Department of Biology of the University of Washington. Larvae were reared on artificial diet (Bell and Joachim, 1976) under long-day light:dark (LD) regimen (LD 17:7) at 25–26°C and 40–50% relative humidity (RH), and prepared for experiments 2–3 d after emergence. In preparation for electrophysiological recording, the moth was secured in a plastic tube with dental wax, leaving the head, and antennae exposed. The preparation was oriented so that both ALs faced upward, and the tracheae and sheath overlying one AL were carefully removed with a pair of fine forceps. The brain was superfused slowly with physiological saline solution throughout the experiment.

Electrophysiological recordings were made with 16-channel silicon multielectrode recording arrays (a4 × 4–3 mm 50–177; NeuroNexus Technologies, Ann Arbor, MI, USA). This microprobe allows the recording of neurons throughout the AL because of the probes dimensions, with four shanks spaced 125 μm apart, each with four recording sites 50 μm apart (Christensen et al., 2000; Riffell et al., 2009b). The probe was positioned under visual control using a stereo microscope. We use routine histological methods (e.g., Riffell et al., 2009a) to visualize the tracks left by the probes and identify the recording sites. Neural ensemble activity was recorded simultaneously from the 16 channels of the recording array using a RZ2 base station (Tucker-Davis Technologies, Alachua, FL, USA) and a PZ2 peamplifier. Spiking data from 16 channels (recorded at four sites on each of the 4 probes) were extracted from the recorded signals and digitized at 25 kHz using the Tucker-Davis Technologies data-acquisition software. Spike data were extracted from the recorded signals in the tetrode configuration and digitized at 25 kHz per channel. Filter settings (typically 0.6–3 kHz) and system gains (typically 5000–20,000) were software adjustable on each channel. Spikes were sorted using a clustering algorithm based on the method of principal components (PCs). Only those clusters that were separated in three dimensional space (PC1–PC3) after statistical verification (multivariate ANOVA; *P* < 0.05) were used for further analysis (6–15 units were isolated per ensemble; *n* = 11 ensembles in as many animals). Each spike in each cluster was time-stamped, and these data were used to create raster plots and to calculate the instantaneous firing-rates (iFRs). Based on the spiking activity, recorded spike trains were identified as an LN or PN (as in Brown et al., 2004; Riffell et al., 2009a, 2013; Lei et al., 2011). All analyses were performed with Neuroexplorer (Nex Technologies, Winston-Salem, NC, USA), or MATLAB (The Mathworks, Natick, MA, USA), using a bin width of 5 ms, unless noted otherwise.

Olfactory stimuli were delivered to the preparation by pulses of air from a constant air stream were diverted through a glass syringe containing a piece of filter paper bearing floral odors. The stimulus was pulsed by means of a solenoid-activated valve controlled by the acquisition software (Tucker-Davis Technologies, Alachua, FL, USA). AL neurons were stimulated with two pairs of odorants: (1) pair: “A”: β-myrcene, a plant-derived odorant used to attract moths (Riffell et al., 2009a), “B”: E10,Z12-hexadecadiennal (bombykal {Bal}), the primary component of the conspecific females sex pheromone (Tumlinson et al., 1989, 1994). (2) pair: “C” BEA-benzaldehyde, and “D”: BOL-benzyl alcohol. Stimulus duration was 200 ms, and five pulses were separated by a 10 s interval. The stimulus durations reflect the time periods in which moths encounter odors when flying in their natural environment (Murils and Jones, 1981; Riffell et al., 2008), and the odorants used to stimulate the preparation are behaviorally effective stimuli, thus allowing neurobiological experimentation in a naturalistic context for discovering how neural circuits process odor information.

## 3. Results

**Figure 1:**
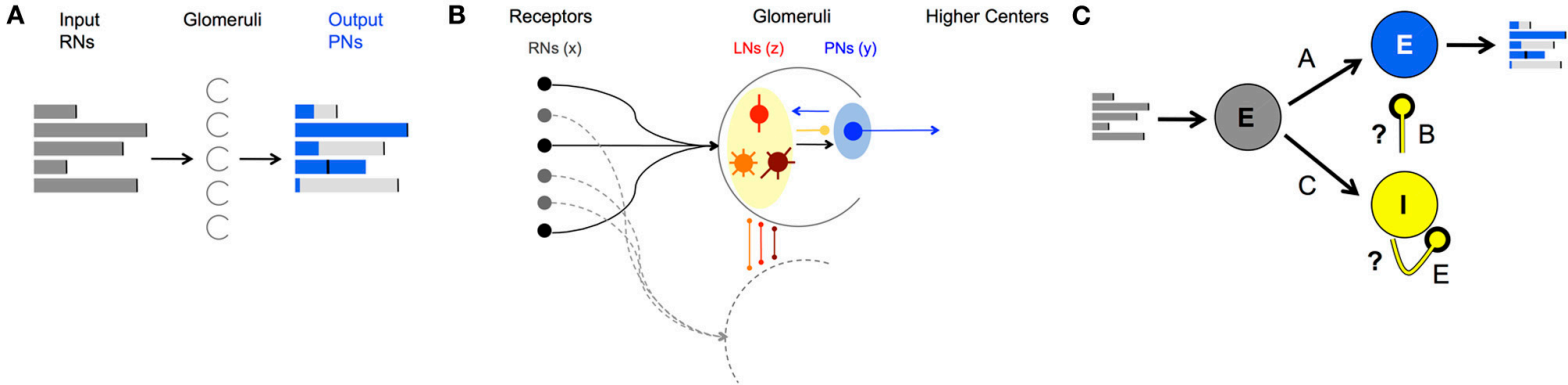
**AL network structure and function. (A)** Demonstration of a neural code: FRs of the input neurons (gray bars) are processed by neural dynamics in the Glomeruli (half circles) in the AL and result with “shaped” FRs of the output neurons (blue bars). **(B)** Anatomical structure of neuron types and wiring in the AL. Both PNs (blue shaded) and LNs (yellow shaded) receive input from RNs (black balls). LNs synapse to LNs and PNs in other glomeruli via local (red), global homogeneous (orange)/heterogeneous (brown) with mainly inhibitory synapses. PNs as output neurons have excitatory synapses to neurons outside the AL, the mushroom body. **(C)** Schematics of a network that mimics the wiring in the AL in moths divided into three populations: input excitatory RNs (gray), interneurons inhibitory LNs (yellow) and projection excitatory PNs (blue). A, B, C, and E denote the connectivity matrices between different populations of neurons, i.e., the connectome. With correct calibration of the inhibitory connections, marked by the question marks, the network can produce neural codes as in **(A)**.

**Figure 2:**
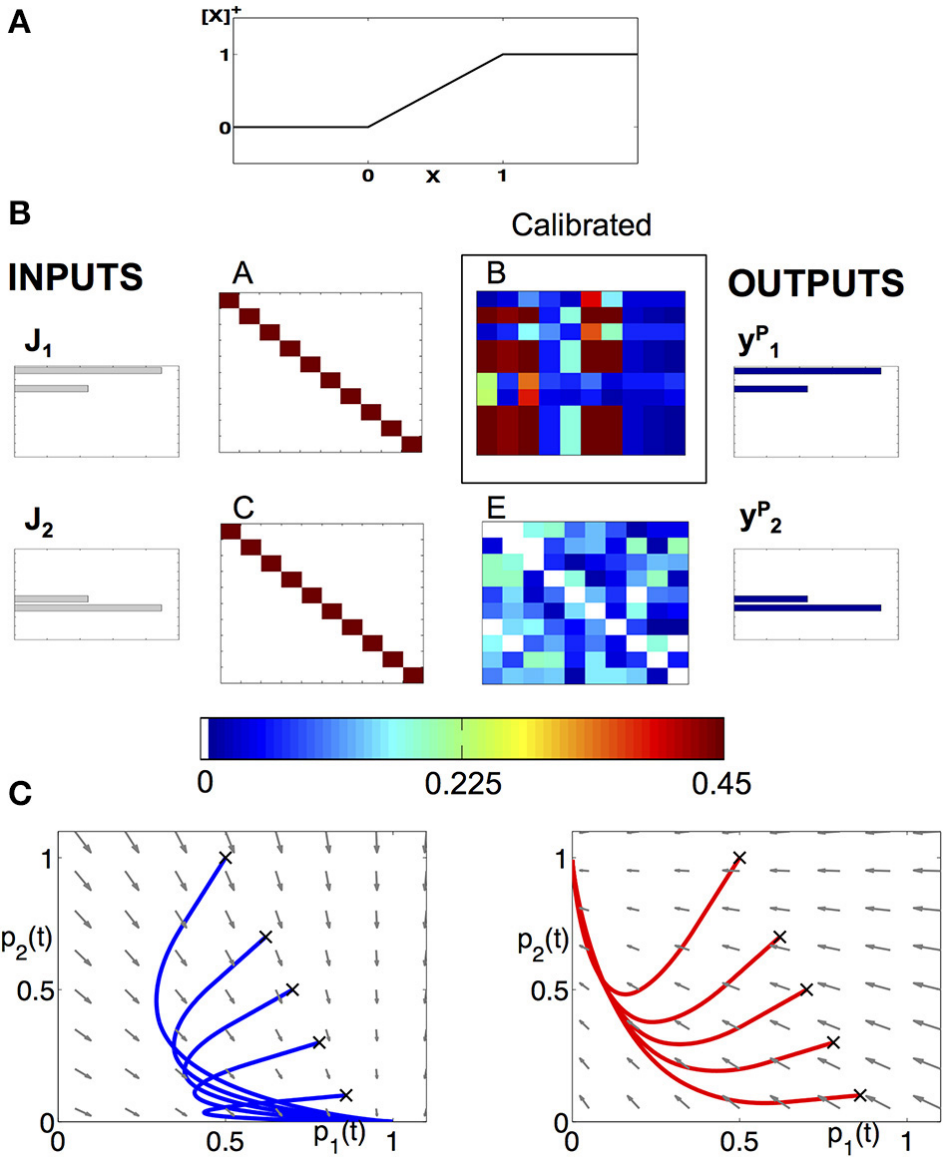
**Modeling approach to the dynamics of the AL. (A)** Piecewise linear f-I curve that we use to model the neurons as FR units. **(B)** Example of a calibration of a network, consisting of 10 neurons from each class and encodes two neural codes. The input keys ${\vec{J}}_{1}$, ${\vec{J}}_{2}$ and the population vectors ${\vec{y}}_{1}^{P},{\vec{y}}_{2}^{P}$ with given connectivity matrices *A, C, E* allow us to reconstruct the matrix *B*. We use the matrix $W=\left[\begin{array}{ccc} 1 & -0.4 & 0\\ -0.4 & 1 & 0\\ -1 & -1 & 1 \end{array}\right]$ to prescribe the low-dimensional projected system. **(C)** Deterministic dynamics projected to (*p*_1_,*p*_2_) plane, with the input being the key: ${\vec{J}}_{1}$ (left) or ${\vec{J}}_{2}$ (right). When ${\vec{J}}_{1}$ is applied, the fixed point is located at (*p*_1_, *p*_2_) = (1,0) and all trajectories are attracted to it (see blue sample trajectories with initial conditions denoted by “x”). When ${\vec{J}}_{2}$ input is applied, the trajectories are being attracted to (*p*_1_, *p*_2_) = (0,1) (see red trajectories).

To study the AL's neural encoding dynamics we computationally model the AL as a network with each neuron modeled as a FR unit. In keeping with the populations of AL cells, three populations of FR units are considered: RNs that carry the input from the periphery (RNs), projection (output) neurons (PNs), and local inhibitory interneurons (LNs) (Figure 1). The dynamics of the populations are represented by the state vectors, $\vec{x}$, $\vec{y}$, and $\vec{z}$ corresponding to dynamics of RNs, PNs, and LNs, respectively. Each FR unit in each population is modeled by a differential equation that describes unit's self-dynamics (decay in the absence of input), interaction with other units and response to odorant stimulus (for a detailed description of the construction see the Materials and Methods section). The network can be calibrated to perform encoding functions, i.e., produce neural codes. Specifically, for each FR pattern that the PNs population exhibits (called *population encoding vector*), there is a FR pattern of the RNs population that evokes it (called *input key*) (depicted in Figures 1, 2). The results that we obtain from constructing the network establish how neurons' connectivity and network dynamics are linked together to produce these encoding functions. Analyzing computational dynamics and comparing them with experimental dynamics elucidates what are the typical dynamics of neural codes and how they can be perceived. We describe our results in detail below.

### 3.1. Recovering the connectome of an example network

As an illustrative example of the theoretical construct proposed here, we demonstrate how we establish the neuronal wiring on a network of 10 neurons of each type: 10 RNs, 10 PNs, and 10 LNs for a total of 30 neurons. The network is designed to encode two input keys into two output population encoding vectors (codes) identical to the input keys. The goal of the calibration is to determine the connectivity matrix *B* given the matrices *A, C*, and *E* (Figure 1C). Specifically, we choose the matrices *A* and *C* to be identity matrices, i.e., each receptor is connected to its corresponding PN and LN. The matrix *E* is set as a random matrix whose elements are drawn from a uniform distribution with mean 0.25, i.e., the LNs are randomly connected between themselves. We then solve an optimization problem, Equation (11), derived in the Materials and Methods section, to determine the elements of the matrix *B*. This is the optimal matrix that supports such an input-output relation (Figure 2B). The matrices are asymmetric, showing that our approach is consistent with experimental anatomical data. Moreover, it is fundamentally different than the Hopfield-type approach that uses symmetry constraint for optimization (Hopfield and Tank, 1986; Reisenman et al., 2008).

**Figure 3:**
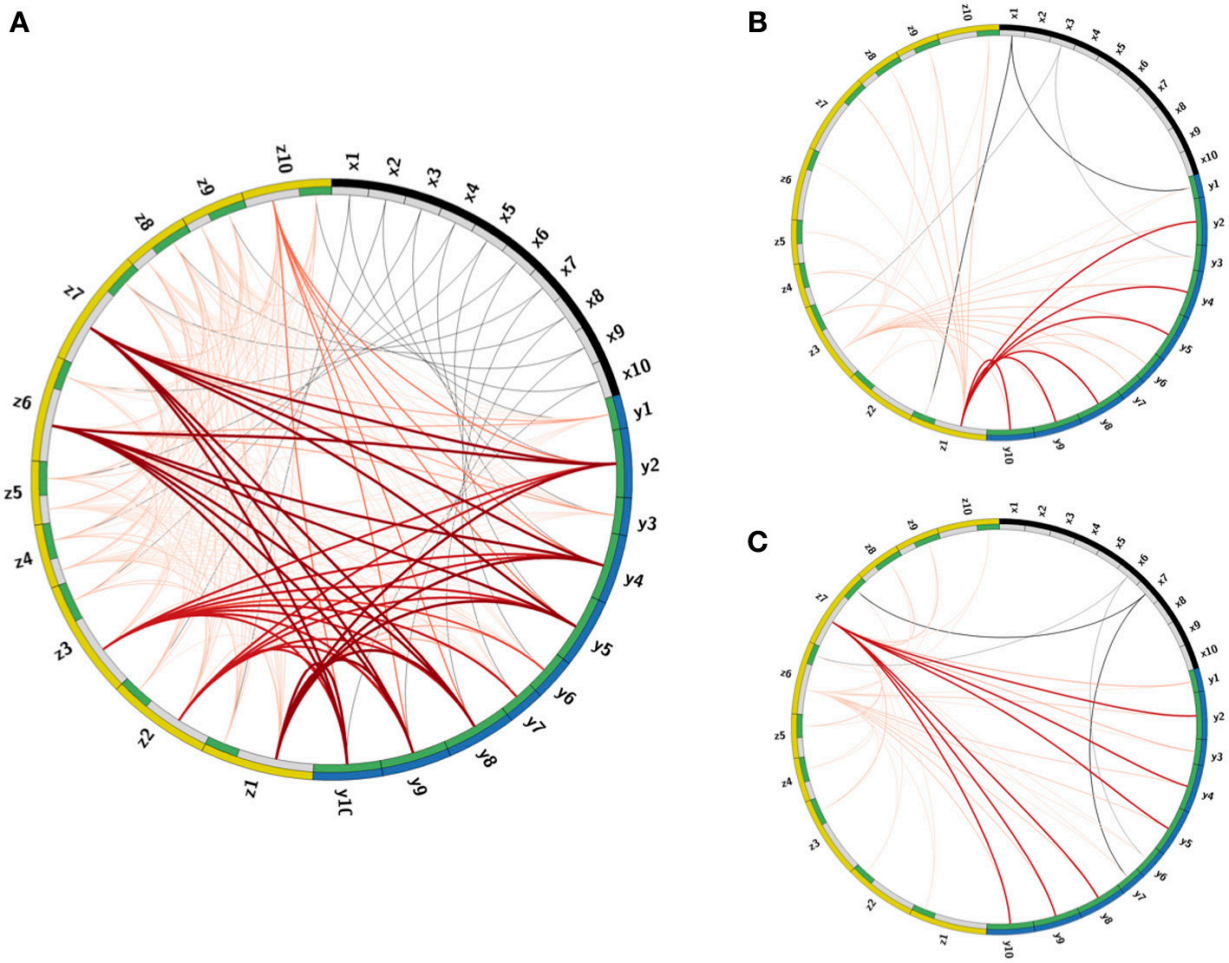
**Visualization of the Connectome. (A)** The connectome (neurons and their connections) of the reconstructed network depicted in a ring shape. The nodes on the outer ring correspond to RNs, LNs, and PNs and marked by black, yellow, and blue colors, respectively. The inner ring splits each node into “in” and “out” terminals colored by gray and green colors. The edges denote connections between the nodes. Excitatory connections are displayed by gray color and inhibitory by red color. The shades and width of the edges denote the strength of the connections (darker and wider curve corresponds to a stronger connection). **(B,C)** Activated pathways in the connectome when the input is the key: ${\vec{J}}_{1}$ **(B)** or ${\vec{J}}_{2}$ **(C)**. The shades and width of the “out” edges in the connectome scaled by the input into the node. When there is no input into the node, its “out” edge is not shown.

The calibration process produces connectivity matrices from which the connectome of the full network is recovered. To visualize the connectome we use the CIRCOS package (Krzywinski et al., 2009) where the network is depicted in a ring shape: FR units are drawn as arcs on the ring's perimeter and the connections are the links between the arcs (Figure 3). The connectome structure allows us to observe that indeed the remainder PNs, labeled as (*y*2, *y*4 − *y*6, *y*9, *y*10), have stronger input inhibitory connections (dark bold red curves) than the PNs that participate in the output codes, labeled as (*y*1, *y*3, *y*6, *y*7). We further observe that these strong connections are output connections of LNs, activated by RNs participating in one of the keys, labeled as (*x*1, *x*3, *x*6, *x*7). This confirms that the strong inhibition of the remainder PNs is activated only when there is enough input from RNs participating in the keys. In addition, each input key activates the suppression of the other key, though less strongly than the suppression of the remainder. This is expected from the calibration matrix *W* specification (see caption of Figure 2, and the definition of *W* in Equation (10). The random connections between the LNs, defined by the connectivity matrix *E*, are seen in the graph as edges marked by light red color.

Once the connections are determined, the deterministic dynamics of the calibrated connectome defined in Equations (1–3) can be explored computationally in order to verify that the calibration gives the desired low-dimensional dynamics. In Figures 3B,C we depict the *active pathways* in the connectome, i.e., the pathways activated by the input keys. We demonstrate that for the input ${\vec{J}}_{1}$ (Figure 3B) four excitatory edges are activated in the connectome, where the edges from *x*1 are stronger than from *x*3 as expected. These edges excite LNs that activate inhibitory pathways to PNs. The strongest inhibitory pathway is invoked by *z*1 that suppresses strongly all remainder PNs. There is also relatively strong suppression of the PNs that participate in the input key ${\vec{J}}_{2}$ and very weak suppression of PNs that should be activated when the input is ${\vec{J}}_{1}$. For the input key ${\vec{J}}_{2}$ (Figure 3C) the remainder is strongly suppressed again, but by a different LN (*z*7). Moreover, the suppression of neurons that should respond to ${\vec{J}}_{1}$ is stronger than that of ${\vec{J}}_{2}$, i.e., the suppression is switched as expected to support ${\vec{J}}_{2}$ instead of ${\vec{J}}_{1}$.

From the structure of the effective connectome, we can conclude that it indeed produces the expected *low-dimensional* dynamics. Further verification is shown in Figure 2C where the dynamics of the full network are exactly the dynamics of the prescribed projected low dimensional system, Equation (7). When ${\vec{J}}_{1}$ is the input, Figure 2C (left), all trajectories are attracted to a unique stable fixed point on the vertical axis, and when the input is ${\vec{J}}_{2}$, Figure 2C (right), the trajectory is attracted to the unique stable fixed point on the horizontal axis.

### 3.2. Noisy inputs

Input into the AL varies significantly as a function of time due to environmental effects, producing low signal-to-noise ratio input signals to the AL. We can use the example network as a prototype system to study the stochastic dynamics of such networks and the implications on the calibration proposed here. To simulate noisy inputs, we define the input as $\vec{J}=\alpha {\vec{J}}_{k}+\sigma \eta (t)$ and define the signal-to-noise ratio (SNR) as α/σ. The noise η(*t*) is modeled as white noise with positive normal distribution, η(*t*) ~ |
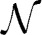
(0,σ)|, and accounts in most general way for the overlap between the stimuli, overlap between RN response and other effects such as spontaneous activity, and channel noise. From recordings when the stimulus was absent or when control stimulus (mineral oil) was applied we estimated that σ = 0.3.

Our objective is to verify that for different SNR ratios, the performance of the network produces the correct population encoding vector, as observed in experimental studies of the AL. To quantify the contrast enhacement, we introduce the measure, contrast over time (CRT), for a noisy input key ${\vec{J}}_{k}$, defined as $CRT_{k}=p_{k}(t)-\sum_{j=1,j\neq k}^{l}p_{j}(t)-r(t)$. This describes the difference between the *k*-th population encoding vector, *p_k_*(*t*), and the summed dynamics over all other population encoding vectors, *p_j_*(*t*), and the remainder *r(t)*.

**Figure 4:**
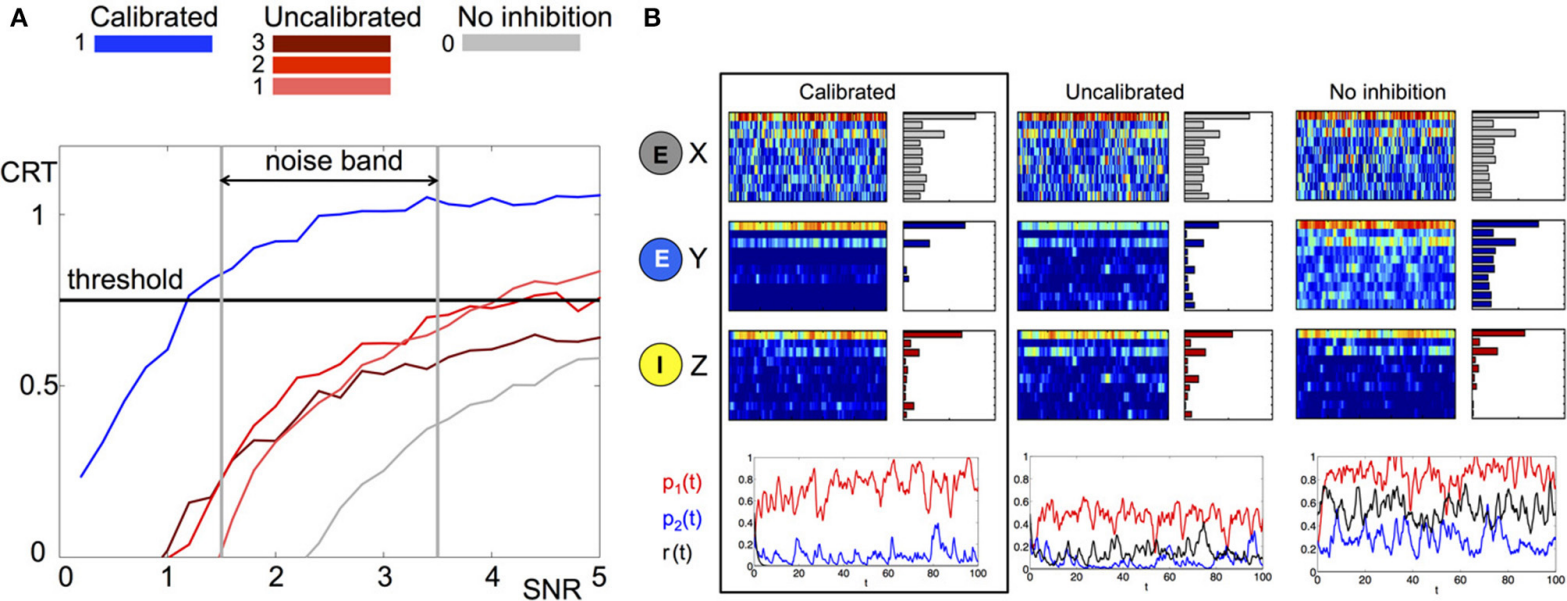
**Comparison of stochastic dynamics of an example network calibrated as in Figures 2B,C. (A)** Contrast (CRT averaged over 10 runs) vs. SNR for different choices of the connectivity matrix *B*: calibrated by (11) (blue), elements drawn from a random uniform distribution with *m*×0.5 mean: *m* = 1(pink), *m* = 2 (red) and *m* = 3 (brown), connections are blocked *B* ≡ 0 (gray). **(B)** Dynamics of a network with noisy input ${\vec{J}}_{1}$ (signal-to-noise ratio SNR = 3) for different choices of the connectivity matrix *B* (from left to right): calibrated by (11), elements drawn from random uniform distribution with mean 0.5, connections are blocked *B* ≡ 0. The elements of the matrix *E* are drawn once from a uniform distribution and fixed. The color raster plots show the FR dynamics of the neurons in X (RNs), Y (PNs), and Z (LNs) classes (blue:low FR, red:high FR). The bar plots on the right side of each raster plot show the average FRs over the whole evolution. The bottom plots show the projection of Y neurons onto the patterns ${\vec{o}}_{1}^{P}$,${\vec{o}}_{2}^{P}$ and ${\vec{o}}^{R}$ corresponding to *p*_1_(*t*), *p*_2_(*t*) and *r(t)* and depicted with *red, blue*, and *black* colors, respectively.

Intuitively, the measure will be larger when there is a better separation between the correct input and all other possible inputs. We investigate the average CRT over time vs. SNR in Figure 4 (left) for three different network structures where the matrix *B* is *calibrated, uncalibrated* (random with different magnitudes) and has *no inhibiton* (all zeros). It can be clearly observed that the calibrated network achieves the best CRT out of all other network wirings. The calibrated network exhibits a 1.5 to 4-fold increase in CRT values in comparison to its corresponding uncalibrated networks, and a 10-fold increase over the case where there is no inhibition. In particular, network calibration is important at low SNR rations (1.5–3.5), which is the expected noise band in the actual environment (Bhandawat et al., 2007; Riffell et al., 2008, 2009b). Otherwise, the correct population encoding vector cannot be separated from the background noise, Figure 4A. Indeed, only the calibrated CRT curve is able to cross the 0.75 CRT threshold (approximately 75% of separation) in that SNR band. By varying the amplitude of the uncalibrated connections we illustrate that the amplitude of the elements in B do not necessarily improve the CRT. When the amplitude is low (see gray curve for 0 amplitude) the performance is poor because the activity is noisy. Incrementally increasing the amplitude improves the performance such that it is able to cross the CRT threshold when SNR exceeds 4 (red curves). However, for a calibrated network the crossing of the threshold happens for much lower values of SNR. Remarkably, even for SNR lower than 1 (where noise prevails over the signal) the calibrated CRT curve (blue) is already crossing the threshold. Additional increase in the amplitude of the inhibitory connections will inhibit both noise and the signal, and we indeed observe that the CRT curve (brown) drops lower than the lowest amplitude curves and does not cross threshold in the 0-5 SNR band.

To understand the contrast enhancement more intuitively, we show in Figure 4B the dynamics for SNR = 3. At this SNR, the dynamics of RNs and LNs are very similar for all network wirings. The dynamics of RNs are noisy, making it very difficult to recover the input key from the data. The dynamics of LNs are cleaner, but still do not have a clear signature of the input key signal. In particular the ratio between the two elements of the key, neurons 1 and 3, is incorrect. The dynamics of the PNs, however, are very different for the three choices of network wirings. In the calibrated network the dynamics of PNs are more distinguishable relative to other networks. Indeed, both FRs over time and average FRs indicate that the output signal is the closest to the population encoding vector *o^P^*_1_ corresponding to the input key ${\vec{J}}_{1}$ (the CRT value is around 1). For uncalibrated or no lateral inhibition wirings, such a clear signature cannot be detected. Indeed the CRT measure for uncalibrated network is 0.55 and for no inhibition network is 0.2.

We also compared the calibrated and uncalibrated wirings obtained from data (stimuli C and D) by adding noise of σ = 0.3 (SNR = 3) to the stimuli and computing the CRT trajectory over time for each simulation (5 simulations per wiring), see Figure S1. Indeed, the CRT trajectories produced by the calibrated model cross the correct threshold, i.e., they approach the correct fixed point, while trajectories produced by the random model do not cross it. In addition, when the stimulus was turned off the trajectories produced using randomly wired model became very sensitive to noise to the extent that they can cross the wrong thresholds.

### 3.3. Construction of the data-driven model

We proceed and construct a dynamical model using the experimental data. In the first series of experiments we recorded from 130 PNs that were stimulated with two odorants: “A” (BAL-Bombykal), “B” (MYR-β-Myrcene). These stimuli are behaviorally effective odorants: odorant A is a component of the moth sex pheromone, and odorant B is a flower scent component. These odorants excite distinct glomeruli in the AL [male sex pheromone is processed in a distinct area—the macroglomerular complex MGC (Christensen and Hildebrand, 1988; Homberg et al., 1988; Hansson et al., 1991; Hildebrand and Shepherd, 1997)] and thus require a minimal orthogonalization of the library. Therefore, we chose them to validate the data-driven model construction part of our approach. Another reason for the choice is that they are (negatively) correlated with each other—when a particular stimulus is on, PNs associated with it are excited while those associated with the other stimulus are inhibited (see Figure S1). This suggests that these regions inhibit each other via lateral inhibition. We also recorded from 77 PNs with two related stimuli: “C” (BEA-benzaldehyde), “D” (BOL-benzyl alcohol) that excite PNs in overlapping glomeruli. Both odorants are dominant in floral scents related in chemical structure as oxygenated aromatic volatiles.

The odorants are presented to the preparation at a realistic time interval (200 ms) repeatedly for five stimulations separated by long intervals of no input. For more information regarding the experimental setup and procedures see the subsection “Electrophysiological preparation and stimulation” in the Materials and Methods section. The data is available in the Supplementary Material. With the spike trains of each PN we have computed the time series of the instantaneous FR (iFR) averaged over the 5 trials of odor introduction. Sampling the iFR at a specific time after the beginning of the odor introduction (at 150 ms) or performing a PCA reduction and taking the first dominant mode, we obtained a histogram of iFRs for the neurons for each of the odors. The neurons with substantial difference in iFR in response to the two odorants were assigned as selective neurons (37 neurons for A,B and 32 neurons for C,D). Those with low iFR were assigned as remainder neurons (60 neurons for A,B and 45 neurons for C,D). The remaining neurons that exhibited high iFR were not included in the calibration (33 neurons for A,B) since there was not enough data to calibrate the inhibitory connections to them.

**Figure 5:**
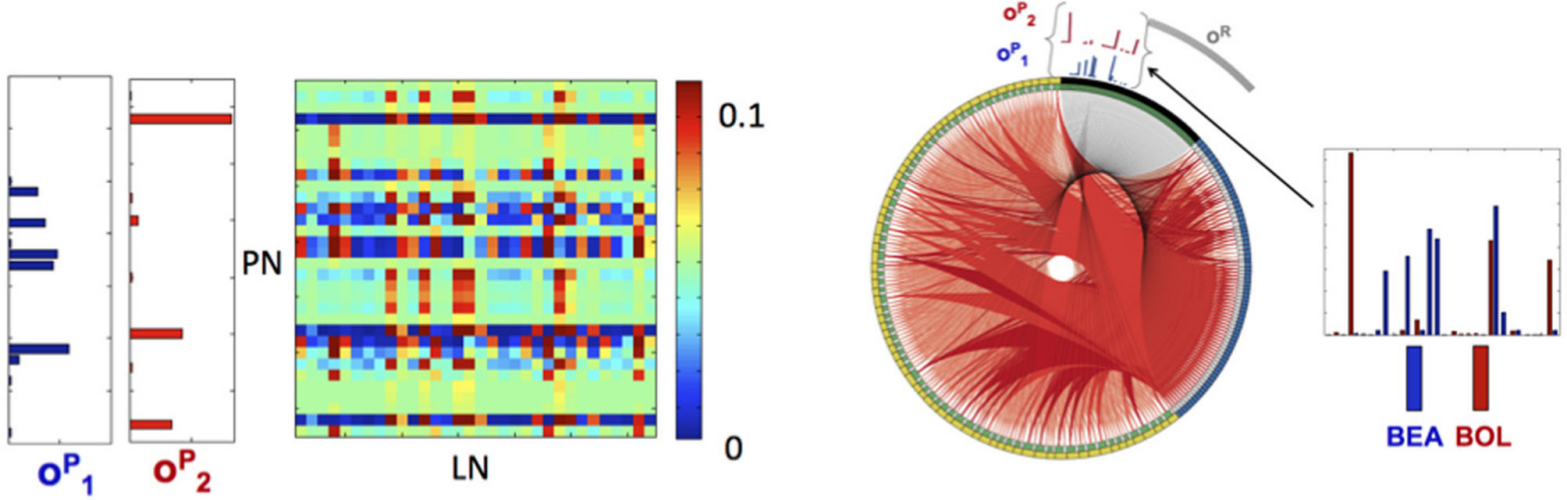
**Library elements and the reconstructed connectome produced from experimental data. Left:** Part of the connectivity matrix *B* connecting LN neurons with active PN neurons (PN-rows, LN-columns) inferred using Equation 11 for patterns that correspond to BEA and BOL (C and D) stimuli. **Right:** The connectome of a network of 77 neurons of each type (231 in total), obtained from solving the minimization problem in (11) with the matrices *A, C* chosen as identity matrices and *E* as a positive random matrix with elements drawn from a normal distribution, i.e., *e_ij_* ~ |*N*(0,0.02)|. The connectome is visualized in a ring shape with similar choice of colors as in Figure 3A. The orthogonal library matrix, *O*, used in the minimization, was obtained from the electrophysiologically recorded data of two odorants BEA and BOL (C and D) as described in subsection Construction of the Data-Driven Model in Results section. The three library vectors ${\vec{o}}_{1}^{P}$, ${\vec{o}}_{2}^{P}$ and ${\vec{o}}^{R}$ in *O*, correspond to the odorants C (blue), D (red) and the remainder (gray), respectively, are depicted section as bar-plots above the connectome at the locations that correspond to RNs that evoke each of the vectors. To the right of the connectome, the bar-plots of the vectors ${\vec{o}}_{1}^{P}$ and ${\vec{o}}_{2}^{P}$ are enlarged. The threshold value, τ, used to construct the library vectors, is τ = 0.07.

Application of the orthogonalization procedure, defined in the Materials and Methods section, for the 97 neurons for A,B and 77 neurons for C,D resulted in the two population encoding vectors: ${\vec{o}}_{1}^{P}$ for C (blue) and ${\vec{o}}_{2}^{P}$ for D (red) as shown in Figure 5 (vectors for A,B are shown in Figure S4). For A,B the required orthogonalization is minimal, while for C,D it is significant as shown in Figure S2.

This allows for the reconstruction of the *connectome* of the AL network in a similar fashion to the example network. Here the matrix *E* is taken as a random normal matrix and the matrix *B* is calibrated. The full network consists of the three populations (PNs, LNs, RNs) of 77 neurons (231 neurons in total), where in each population we depict (in the clockwise direction) the selective neurons followed by the remainder neurons (active submatrix *B* Figure 5 left and connectome Figure 5 right). Although many connections exist, the ring shaped visualization demonstrates qualitatively the main features of the connectome: (1) the suppression of the selective neurons seems to be non-uniform and sparse while (2) the inhibition of remainder neurons is uniform and dense. The non-uniformity of the wirings is consistent with the non-uniformity of the population encoding vectors.

### 3.4. The dynamics of population encoding vectors

**Figure 6:**
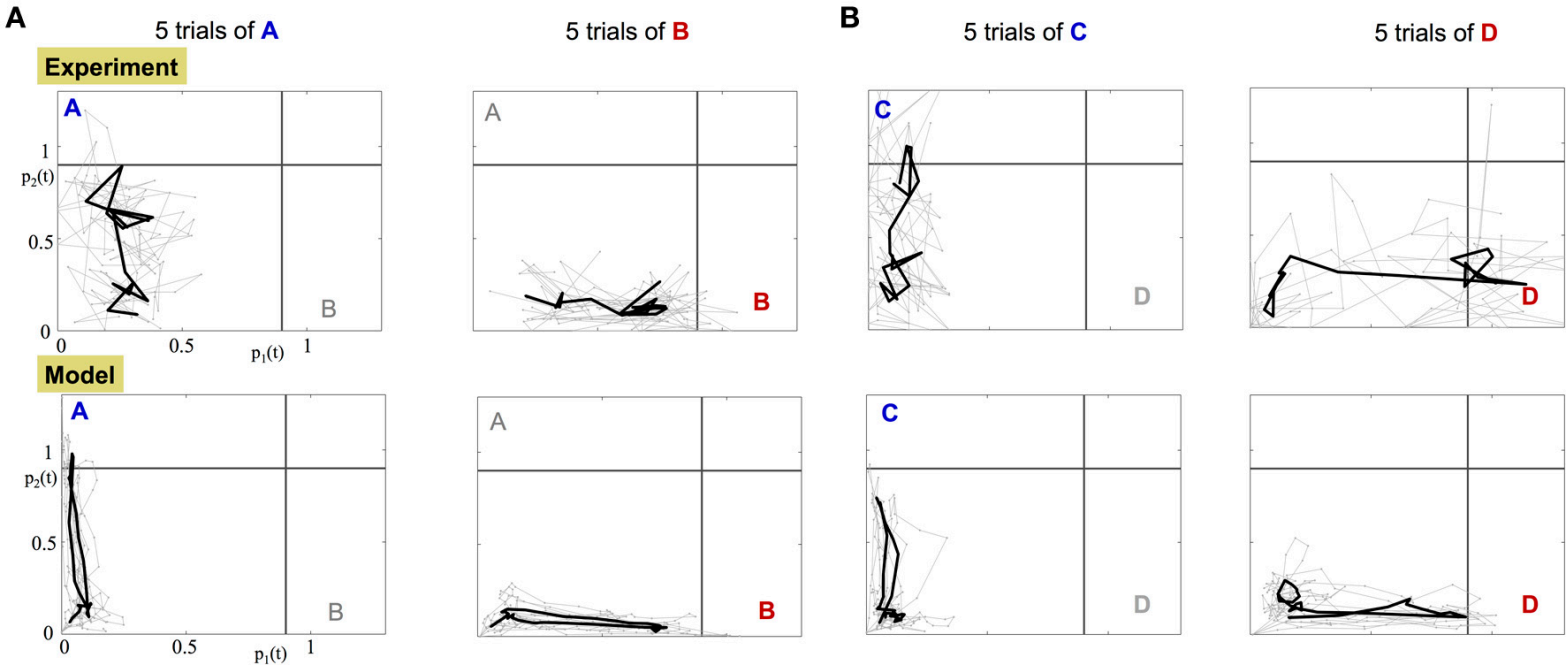
**Projection of the experimental and model dynamics onto orthogonal odor space. Top row:** projected experimental dynamics of PNs. **Bottom row:** projected dynamics of the calibrated model. Each column shows 5 distinct trials per stimulus; **(A)** for A,B stimuli; **(B)** for C,D stimuli. Gray trajectories are 5 distinct trials of the application of the odor. The starting and ending points of the plotted trajectories are 100 and 600 ms, respectively, after the beginning of the trial. The black bold trajectory is the averaged trajectory over the trials.

The orthogonality of the population encoding vectors, ${\vec{o}}_{1}^{P}$ and ${\vec{o}}_{2}^{P}$, allows us to construct a two dimensional space, called the *odor space*. We use it to project the iFR time series obtained from either the data or the calibrated model. The data projection is used to assess whether the experimental dynamics are consistent with the underlying dynamical mechanism in the construction of the model. Specifically, there is a single, stable fixed point in each encoding vector direction. Figure 6 shows the projection dynamics (gray) of five experimental trials along with the average trajectory over the trials (black). Before the input is applied, the projected trajectory hovers around the origin due to noise fluctuations. When the input is applied, the trajectory begins an excursion toward the stable fixed point and when the input is off, the trajectory returns to within the vicinity of the origin. The input of odor “A,” “C” corresponds to trajectories whose fixed point lies on the vertical axis while odor “B,” “D” trajectories evolve trajectories toward a fixed point on the horizontal axis.

Data projections from both the experimental data and the calibrated model, Figure 6 and Supplementary Videos, clearly demonstrate that different odorant inputs correspond to different orthogonal fixed points in the projection space. Furthermore, trajectories appear noisy while reaching the fixed point whereupon they remain static for a while until the input is stopped and then trajectory returns to the origin.

### 3.5. Decision making

In the experiments described here, the presentation of a stimulus odor occurs for an extremely short period of time (approximately 200 ms). Such inputs correspond to realistic stimulus for which the moth is flying and sampling odors in a turbulent environment. Thus, once we have characterized the dynamics of each short trial, we examine possible classifiers for odor detection and selection.

To formulate the decision making process, we analyze the dynamics of a trajectory toward the orthogonal fixed point when the stimulus is introduced as demonstrated in Figure 6. The orthogonality of the fixed points allows us to construct *threshold lines* for determining odor detection. The gray horizontal and vertical lines in Figure 6 represent the threshold for the detection of odor A and odor B, respectively. Application of a single odorant ensures that the dynamical trajectory crosses only a *single* threshold line on its way to its corresponding fixed point. Experiments show that it spends only a small amount of time near the fixed point (approximately 100 ms) before returning back to the origin.

**Figure 7:**
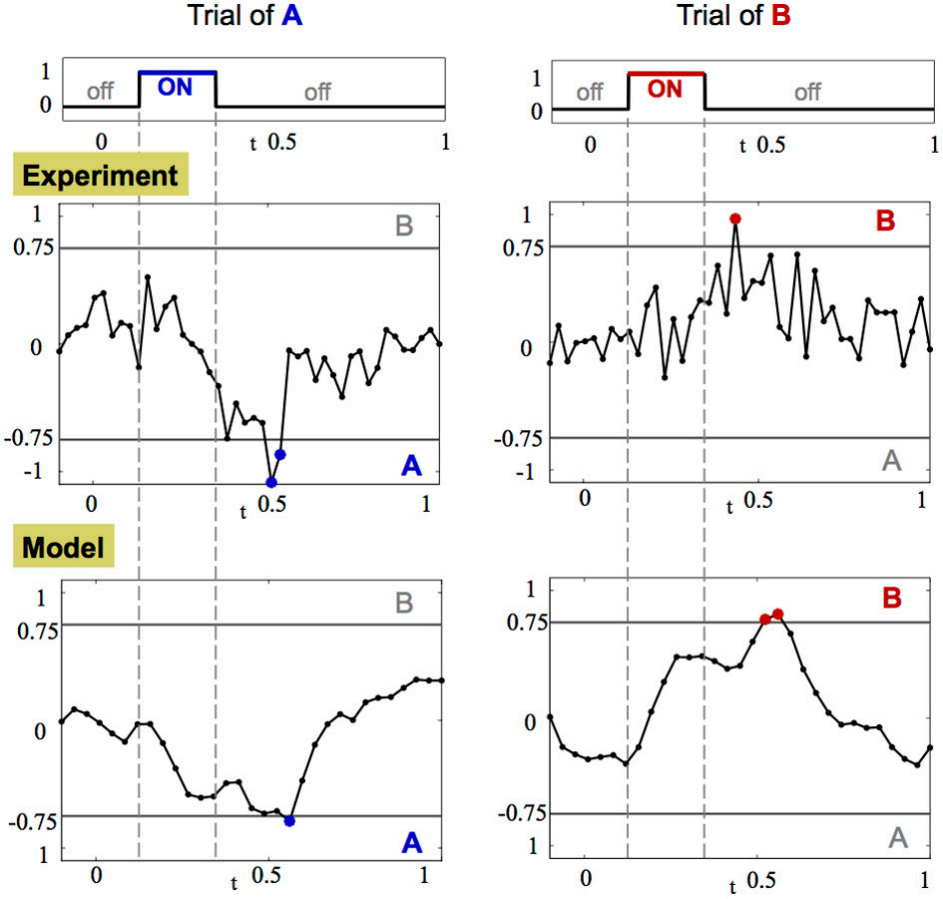
**CRT dynamics demonstrates the decision making associated with odor detection**. Left and right columns show the dynamics of *CRT_A/B_* measure for one trial of stimulus A and B, respectively. The CRT measure computed for stimuli A and B is: *CRT_A/B_* = *p_B_*(*t*)− *p_A_*(*t*) ± *r*(*t*). **Top row:** the amplitude of the stimulus input over time. **Middle and bottom rows**: CRT dynamics in the experiment and in the calibrated model, respectively. The threshold decision lines are plotted with gray color at −0.75 (A) and 0.75 (B). When the CRT curve (black line with dots corresponding to data points) crosses the threshold the data points are marked with appropriate color of their dots: blue for stimulus A and red for stimulus B.

While it is difficult to measure the convergence rate of the trajectory to the fixed point, it is straightforward to detect a crossing of the threshold line. Indeed, a common hypothesis in decision making associates crossing of a threshold in neuronal activity as equivalent to making a decision (Bogacz et al., 2006; Wong and Wang, 2006). The crossing of the threshold line in our case can be captured most effectively by computing the CRT measure, shown in Figure 7, per each trajectory of each odorant. Results from this analysis demonstrate that after the input is introduced, the CRT curve tends toward one of the decision thresholds, passes it and then returns back to the region where no clear contrast exists between odorants. Thereby, passing of the threshold creates an evidence toward one of the odorants. Integration of such evidence over several trials can produce a significant bias toward a specific odorant. When enough crossings from trial to trial occur, strong evidence is accumulated to accurately determine an odorant.

From simulations we observe that there is a clear advantage in repetitive introduction of the input in short bursts rather than a single long input. Long input, when noisy, creates a corresponding noisy trajectory that typically crosses the threshold line a single time and then wanders around the fixed point so that the decision is based only on one evidence. Other measures such as the time the trajectory spent near the fixed point are typically non-robust when noisy dynamics are considered. In contrast, repetitive inputs generate a mechanism that allows for better integration of evidence since for each trial it is enough to just cross the threshold once given by a simple measure like the CRT. Such a mechanism thus provides a rapid, robust approach to odor detection.

## 4. Discussion

In this study we introduce a new method for data-driven model construction of the AL. We validate our construction with two novel extensive real multi-neuron recorded data sets, recorded for several stimuli, both related (overlapping) and orthogonal (pheromone and floral), repeated for at least five trials and applied for realistic time scales. With the constructed model we are able to get valuable insights on the biological questions of *how lateral inhibition is wired* and show that *calibration of wiring is required for contrast enhancement*. While these questions were observed and discussed in the cited literature, (e.g., Laurent, 2002; Cleland and Linster, 2005; Mazor and Laurent, 2005; Rabinovich et al., 2006; Buckley and Nowotny, 2012; Capurro et al., 2012), here for the first time we give concrete answer on how to calibrate the network and the impact of wiring on contrast enhancement. Our results also link between independent previous experimental observations, e.g., contrast enhancement (Christie and Westbrook, 2006; Reisenman et al., 2008) with non-local wiring (Silbering and Galizia, 2007; Riffell et al., 2009a).

Contrasted with previous studies, (e.g., Afraimovich et al., 2004; Cleland and Linster, 2005; Mazor and Laurent, 2005; Buckley and Nowotny, 2012; Capurro et al., 2012), our work introduces several novel components which allow us to construct the data-driven model. The first component is that the construction of the odor space introduced here is optimal and allows for obtaining a discriminative set of orthogonal neural codes in contrast to constructions based on PCA that are limited (Galán et al., 2004; Mazor and Laurent, 2005). This basis allows us to achieve inference of the wiring from data vs. random setup used in previous works (Cleland and Linster, 2005; Buckley and Nowotny, 2012; Capurro et al., 2012). The advantage of the method introduced here is that there is no additional fitting to be done in the model and trajectories of our model, in response to stimuli, show close resemblance when compared directly with data. Prior to our approach, model generated trajectories were theoretical and could not be compared with data on neural level. We then show the important role that calibrated wiring plays in contrast enhancement, which was observed as a phenomenon in previous work but the mechanisms for it were not yet specified (Christie and Westbrook, 2006; Reisenman et al., 2008). To quantify the effect of network wiring on the dynamics of both the model and the data we define the contrast enhancement metric (CRT metric) and show that the CRT performance of the calibrated network prevails random calibrated networks. In addition, to explore the realistic transient dynamics and their relation to olfactory decision making, we apply the stimuli for short realistic times (200 ms) vs. much longer time scales in related work (3–5 s) that showed fixed point dynamics, (e.g., Mazor and Laurent, 2005), and observe that trajectories are still able to approach the fixed points, by crossing their associated threshold lines, both in the model and data.

### 4.1. High-dimensional neural network is tuned to exhibit low-dimensional dynamics

Our primary contribution is the introduction of a new methodology that combines dimensionality reduction of dynamical systems with experimental data in order to achieve a reliable computational model, that highlights the exploitation of low-dimensional encoding in the AL. To our knowledge, this is the first successful model that combines such a data-driven methodology in conjunction with dynamical equations of FR activity.

The methodology is divided into two stages. The first stage is implicit, where we define an (implicit) library of population (encoding) vectors and project the dynamic neuronal network onto these vectors (Sirovich, 1996; Shlizerman et al., 2012). The outcome is then restricted so that the system possesses stable orthogonal fixed points, where each orthogonal direction is associated with a different population encoding vector. This restriction determines a mapping from the high- to low-dimensional system. At this stage, the connectivity is kept general and expressed (implicitly) by the matrices *A, B, C, E*. As such it is generalizable to modeling the AL across different species and other neuronal networks. In particular, a similar approach could be applicable to model networks that are further downstream in olfactory processing such as the mushroom body (MB), a neuronal unit that receives input from the AL and associated with memory and learning of odors. Within the MB, Kenyon cells (KC) receive input from PN neurons and synapse to extrinsic neurons (EN). When recordings from EN become available, a similar construction can be performed to recover the connectivity of KCs to ENs (Froese et al., 2014). Furthermore, such a quasi approach (modeling both the AL and the MB) can provide a data-driven model for testing plasticity of connectivity in the AL and MB and how strategies for learning-induced modulation adjust the odor space representation and the metric for decision making (Faber et al., 1999; Farooqui et al., 2003; Cassenaer and Laurent, 2012; Dacks et al., 2012; Bazhenov et al., 2013). Such modulatory effects on the encoding properties are work for future studies. The model developed here provides an efficient platform for performing such studies.

In the second stage of the modeling, the constructed mapping is used in conjunction with experimental recordings to both determine the population encoding vectors and reconstruct the AL connectome associated with each odor in the library (see Figure 4). The construct is consistent with AL experimentally described functionality: while it is a high-dimensional neural network consisting of thousands of neurons, it appears to be tuned to exhibit low-dimensional coding dynamics.

### 4.2. Model setup and extensions

The model constructed here is based on experimental findings and derivation that showed that dynamics induced by stimuli converge to displaced fixed points in a low-dimensional space (Galán et al., 2004; Mazor and Laurent, 2005; Buckley and Nowotny, 2012). Lateral inhibitory connections (LN to PN), the majority of connections within the AL, are supporting these dynamics (Sachse and Galizia, 2002). Our methodology allows for inference of these inhibitory connections by deriving the minimization problem in Equation (11), where a first order approximation of fixed points is being computed by calibrating lateral inhibitory connections.

The propagation of information in the model is similar to propagation of signals in the AL. Signal propagation in the AL is initiated when the stimulus is turned ON. LN and PN dynamics are then induced by the stimulus input. In LN population, the dynamics respond to the input by releasing two GABA transmitters GABA-A (fast, order of few ms) and GABA-B (slow) (Meyer et al., 2013). The connections that are faster, GABA-A, are modeled by the matrix *E* and correspond to the first order approximation of the fixed point. Indeed, we assume that fast GABA-A dynamics allow for quick trajectory (faster than the single ON duration of the stimulus) from quiescent state to the fixed point. Adding GABA-B connections would be a second order correction for the fixed point and could improve the estimate, however, it is not expected to play a significant role for the time scales of the stimulus (of 200 ms) that we consider. In contrast to LN population, PN population receives two inputs: directly from the stimulus (RN to LN) and from LN (RN to LN to PN), such that the timescale of PN response will be determined by these two inputs. In the model they are controlled by norms of the matrices *A, B* and parameter γ. The pathway that involves LN is longer since LN is an intermediate step to reach PN. Previous works, however, show that AMPA connections in GABAergic systems can be much faster than in non-GABAergic suggesting that the two pathways could be of comparable timescales, as we assume in the calibration of the model (Geiger et al., 1997). Experimental and computational explorations of the effects of different timescales on the dynamics are left for future work.

In addition, excitatory LN to PN and PN to PN connections were found in fruit flies, honeybees, although in much smaller numbers than inhibitory LN to PN (Olsen et al., 2007; Root et al., 2007; Shang et al., 2007; Sinakevitch et al., 2013). Their presence suggests that incorporating these connections would be important in deriving a data-driven generic AL model across species. While related work is ambiguous about the impact of these connections, e.g., see Serrano et al. (2013) where the gain control condition of the AL depended only on the inhibitory connections regardless of inclusion of excitatory connections, in principle, such connections could have an impact on the stability of the fixed points and their dynamics. For example, if one of the fixed points loses stability, the dynamics then would be oscillatory or expressed by homoclinic/heteroclinic orbits (Afraimovich et al., 2004; Rabinovich et al., 2006). Even if all the fixed points remain stable, the transient dynamics could differ, e.g., the trajectories could spiral into the (stable focus) fixed point.

The data-driven construction can be extended to include excitatory connections and these different dynamics. Incorporation of excitatory connections and requiring that the dynamics are of the same features (quick dynamics to a stable fixed point) would add additional conditions to Equation (11). For example, a stability condition would restrict all the eigenvalues of the system in Equation (8) to be negative. This condition can be formulated as a Lyapunov equation, i.e., minimizing for an additional matrix, usually denoted by *Q*, that solves the Lyapunov equation and is positive (Gajic and Lelic, 1996). Furthermore, in a similar manner, by imposing pure imaginary eigenvalues in the low dimensional space, the dynamics could be set to oscillatory around some of the fixed points or unstable when the eigenvalues have real positive components.

### 4.3. Lateral inhibition and contrast enhancement

With this framework established, we are able to suggest answers to key questions in the behavior of the AL. One of primary importance is identifying the optimal network design that maximizes contrast enhancement and reproduces the observed AL functionality. The model shows that the optimal design can be constructed by tuning the lateral-inhibition so that the patterns of FR activity are made robust (Rabinovich et al., 2008). In particular, we show that asymmetric, non-local design of connections in a network of neurons can lead to such low dimensional robust functionality.

Furthermore, we demonstrate that in a noisy environment, network tuning is *necessary* for robust detection of an odor, even when input keys and output codes are identical. We show that lateral-inhibition, that has been tuned, shapes the noisy input into reliable and repeatable trajectories, while inhibition that was not tuned produces noisy and unreliable trajectories. This phenomenon is experimentally observed and described as contrast enhancement. Furthermore, our work suggests that absence of inhibition will result in noisier responses and scattered trajectories in the odor space. These predictions can be verified by pharmacological treatment of the AL with GABA antagonists that block inhibition.

Lateral inhibition is essential for shaping the response to complex stimuli, i.e., a mixture of odorants (Laing and Francis, 1989; Duchamp-Viret et al., 2003). Neural responses to these stimuli were shown to be of non-linear nature and phenomenologically classified into three major types: suppression (when the response to a mixture is lower than of a single odorant), hypoadditivity (response is equivalent to a dominant single odorant) and synergism (responses are magnified) (Capurro et al., 2012). The inferred wiring in our model can permit these various types of dynamics through competition. The responses are controlled via the matrix *W* that specifies the weights of interaction between the neural codes. Higher inhibitory weights for a particular odorant indicates that the response might be of a winner-take-all type (i.e., hypoadditivity) regime. If several odorants are of comparable influence on inhibition then they are most likely to settle to a lower response – mutual existence type and effectively exhibit suppression type responses. For strongly overlapping stimuli we can expect to observe the effect of synergism as well—a blend of odorants can create a stronger effective stimulus into one of the axes of the odor space. Although for achieving a significant synergism excitatory LN-PN or PN-PN connections may play a role. In future studies, simulations of the model and variation of the elements of the matrix *W* (similarly to Capurro et al., 2012 for random connectivity), in conjunction with the decision making algorithm and comparison with experimental trajectories, can reveal the regimes that odor competition produces.

### 4.4. Projection space for odor detection

In previous studies, a three dimensional projection space (*odor space*) was constructed using PCA based dimension reduction. Projections of distinct odorant trajectories onto this low-dimensional space appeared to be well separated from each other (Laurent, 2002; Galán et al., 2004; Mazor and Laurent, 2005). Moreover, for each odor there was an associated fixed point that was separated from all other odor fixed points. The construction demonstrated that odorants can be classified into distinct groups and suggested that odor detection may be accomplished solely from recordings and projection onto the odor space.

The odor space is the backbone of our model as well. There are key differences, however, in the construction of our underlying odor space. Specifically, we treat the data differently by dividing the population of PNs into remainder and population encoding vectors so that we achieve a model representing the dynamics of the spatio-temporal FR patterns rather than single neurons. Such a viewpoint of the data is useful since it constructs an odor space (phase space of a dynamical system) with meaningful axes, i.e., our dimensionality reduction gives an orthogonal basis where each vector corresponds to an individual odorant (Figure S3) or a remainder (Figure 2C). As a result, the odor space provides an easy means for odor recognition and characterization.

### 4.5. Decision making as a robust mechanism for odor perception

The timescales of realistic inputs indicate that odor detection occurs relatively fast and usually requires repetitive (over several trials) exposure to the same odor (Koehl et al., 2001; Mainland and Sobel, 2006). Some animals use sniffing or other mechanisms to achieve fast repetition of similar input into the olfactory system (Mafra-Neto and Carde, 1994; Vickers and Baker, 1994; Mazurek et al., 2003; Riffell et al., 2009b). Furthermore, there exists experimental evidence that shows that for a longer stimulus duration (a few seconds), initial sharp response of PNs is followed by more intermittent one (Christensen and Hildebrand, 1988; Marion-Poll and Tobin, 1992). These results suggest that the optimal strategy for scent recognition is employed by sampling the stimulus. For Manduca sexta, the optimal frequency for behavioral response to the Datura flower appears to be 1 Hz with the stimulus applied for 500 ms in each period (similar to honeybees Wright et al., 2009). Neural responses are observed to reach a maximum fixed point at about 100 ms. These time scales indicate that there is a 5-fold difference between neural and behavioral responses which could be due to repetitive sampling of stimulus.

Our analysis suggests that indeed based on the dynamics of the AL the exposure to multiple, short-time bursts of odor can be formulated as a decision making process. More precisely, we are able to prescribe an algorithm, possibly evoked by higher centers in the brain such as the MB or the lateral horn, that poll the dynamics of the AL in order to make a decision. Examination of the projections of iFR data produced in both theory and experiment indicates that in each short trial, the most plausible dynamical response is an excursion in odor space along a trajectory attracted toward an orthogonal fixed point. In fact, the orthogonality of the fixed points allows for an optimal separation of trajectories for different odors. Due to the short timescale of the odor burst, the trajectory does not necessarily converge to the fixed point. Rather, it only approaches its vicinity (Figure 6 and Supplementary Videos). In effect, it crosses the threshold line of an odorant while staying away from crossing thresholds of other odorants, see the horizontal and vertical lines in Figure 6 and the trajectories that cross them. Indeed, a common hypothesis in decision making is that the decision is made when neuronal activity crosses a threshold (Bogacz et al., 2006). Tracking trajectories that cross decision thresholds is accomplished by defining a linear contrast measure over time as we demonstrate in Figure 7. Repetition of the same odorant stimulus permits robustness of the algorithm. With each threshold crossing, evidence is integrated toward a specific odorant stimulus. After each trial, a probability distribution is updated until there is a high probability that supports a specific odorant stimulus. This indicates that enough evidence was integrated toward one of the odorants, and thus leads to a decision/perception for the odor, which is followed by a behavioral response corresponding to that odorant. The signature of this decision making mechanism is that integration for longer time will cease to improve accuracy. In the context of mammalian olfaction (rats) it was shown that indeed decisions do not necessarily improve with additional time (Zariwala et al., 2013). Future experiments that test behavior for various durations of stimuli may provide more evidence into olfactory decision making and its underlying mechanisms.

The proposed algorithm is scalable and can be used for perception of complex odors, i.e., a mixture of odorants (Laing and Francis, 1989; Duchamp-Viret et al., 2003). If the odorants in the compound are of similar significance and strength, then the trajectories in the odor space may cross several thresholds of distinct odorants each time that a stimulus is applied. Repeating the application of the same stimulus, eventually will lead to reconstruction of a uniform probability distribution indicative of the distribution of odorants in the mixture. Note that to obtain a reliable probability distribution the process may require many repetitions than in the detection of a single odorant.

### Conflict of interest statement

The authors declare that the research was conducted in the absence of any commercial or financial relationships that could be construed as a potential conflict of interest.

## Supplementary material

### Data sharing

Data is shared at: http://dx.doi.org/10.6084/m9.figshare.904819
