# Supplementary material for: Data-driven inference of network connectivity for modeling the dynamics of neural codes in the insect antennal lobe
Source: Front Comput Neurosci. 2014 Aug 13;8:70. doi: 10.3389/fncom.2014.00070 (PMC4131428; doi:10.3389/fncom.2014.00070)
Supplement: Supplementary file 3 [file Presentation1.PDF]

Data-driven inference of network connectivity for modeling the dynamics of neural codes in the insect antennal lobe

Eli Shlizerman · Jeffrey A. Riffell · J. Nathan Kutz

Supplementary Material

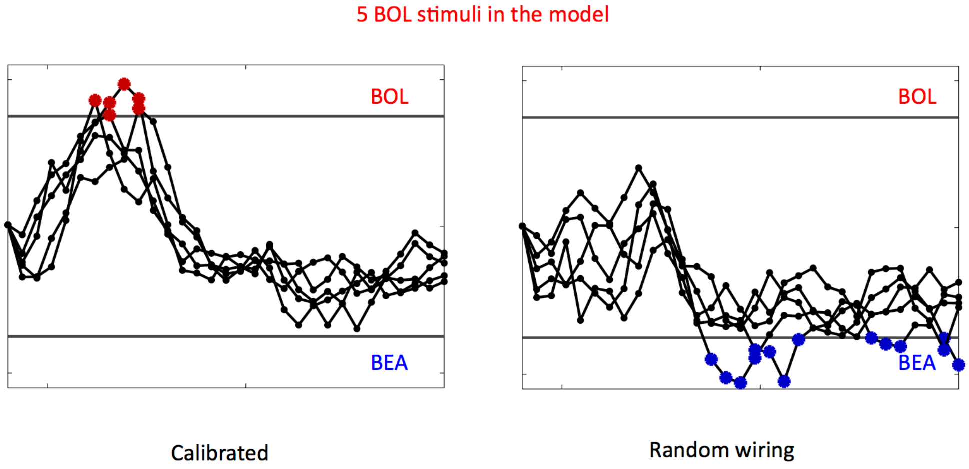

Figure Sup 1: Left: CRT measure for the PN dynamics of a calibrated network stimulated with **BOL** stimulus (positive (red) CRT). Right: CRT measure for a random (drawn from uniform distribution) network.

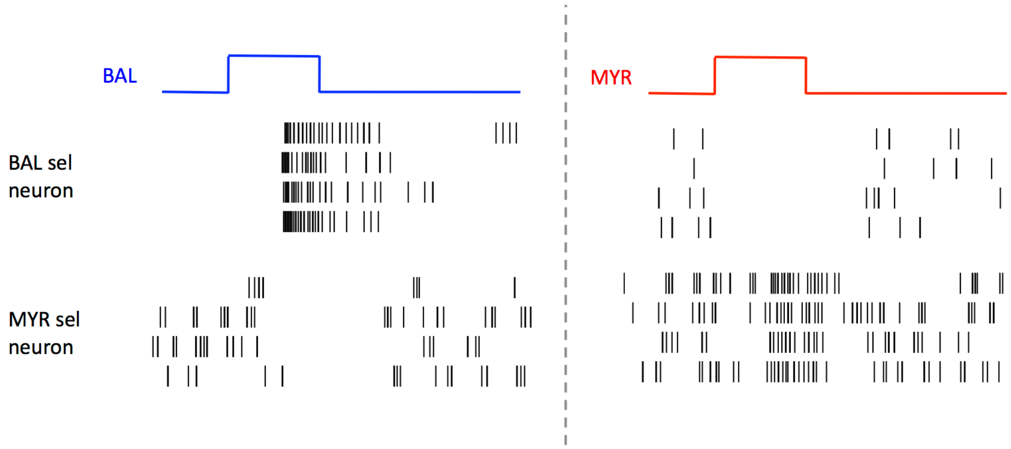

Figure Sup 2: Spike trains of responses of two neurons (top: selective for BAL; bottom: selective for MYR).

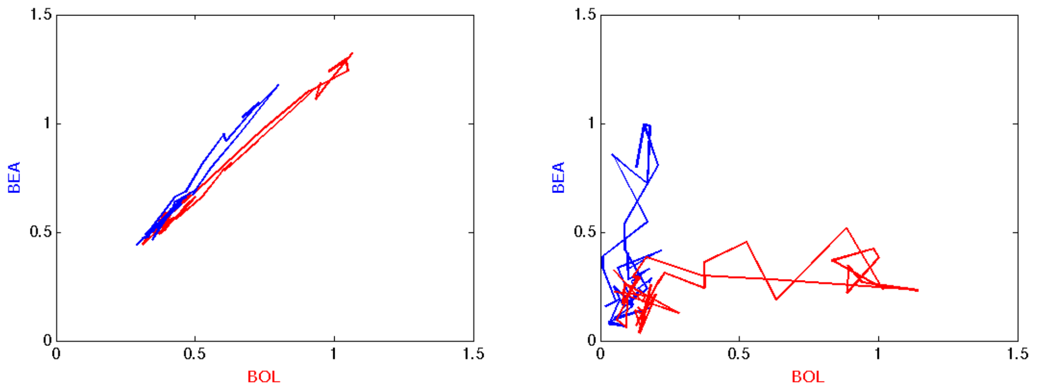

Figure Sup 3: Left: Projection onto the library containing two modes (i.e. per stimulus we take the first PCA mode). Right: Orthogonalization of the library into two orthogonal **distinctive** modes.

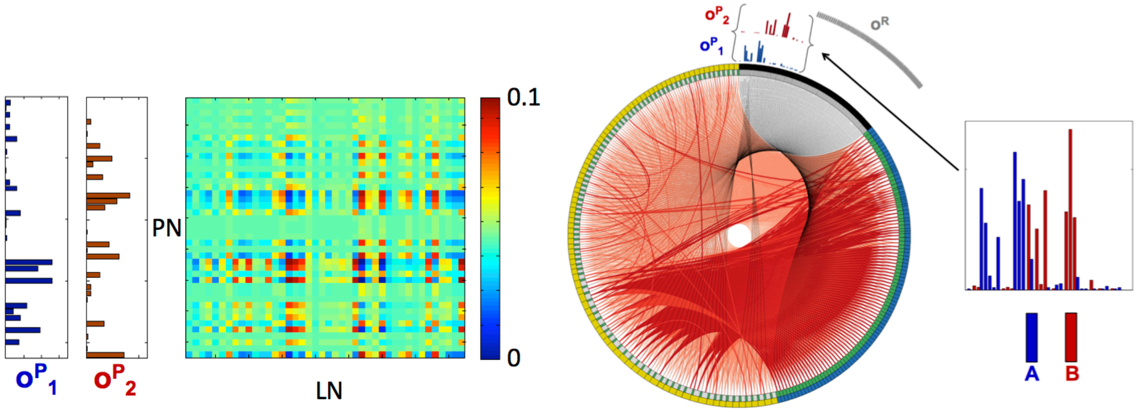

Figure Sup 4: The network constructed for stimuli “A” and “B” (supplementary to the network constructed for “C” and “D” shown the main manuscript, Figure 5). Left: The matrix B connecting LNs with PNs. Right: Network wiring in a polar plot. See Figure 5 for a full description
